# A case of cautery-version

**DOI:** 10.1016/j.hrcr.2021.05.008

**Published:** 2021-05-20

**Authors:** Evan J. Wiens, Colette M. Seifer

**Affiliations:** Section of Cardiology, Department of Internal Medicine, University of Manitoba, Winnipeg, Canada

**Keywords:** Atrial flutter, Cardioversion, Cautery, Pacemaker, Pulse generator

## Introduction

Electrocautery has previously been reported to induce ventricular arrhythmias in rare cases.^1^, ^2^, ^3^ However, to our knowledge, induction or cardioversion of atrial arrhythmias has not been reported. We report a case of repeated cardioversion from atrial flutter to sinus rhythm during electrocautery in a patient with a dual-chamber pacemaker.

## Case

**Figure 1:**
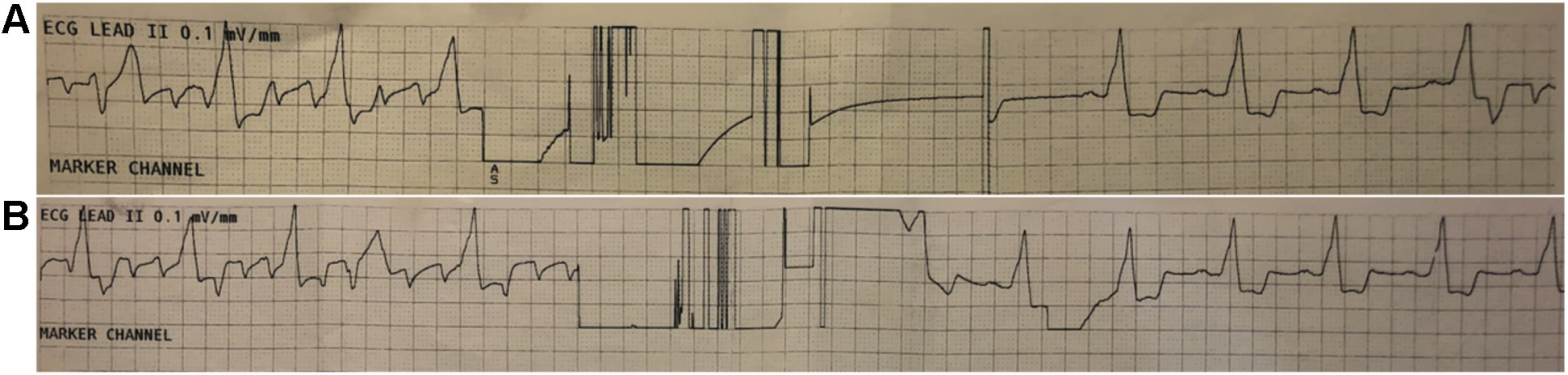
Electrocardiogram. **A:** Rhythm strip showing atrial flutter initially, followed by cardioversion to sinus rhythm with electrocautery. Atrial flutter resumes at the end of the strip. **B:** Subsequent recurrent cardioversion from atrial flutter to sinus rhythm with electrocautery.

A patient with atrial flutter and a Sorin (MicroPort™) pacemaker, a Medtronic™ 4076 (Medtronic, Minneapolis, MN) 52 cm lead in the right atrium and a 4076 58 cm lead in the right ventricle, underwent a pulse generator replacement without interruption of anticoagulation. The device was programmed in DDI mode and both leads were programmed in a bipolar configuration. The device was located in a subcutaneous pocket in a left anterolateral, prepectoral position. During dissection to the device, brief pulses of monopolar cautery at a 2 cm distance from the device were applied. The patient return electrode for the cautery was positioned on the upper left lateral thigh. It was noted incidentally that during cautery the rhythm converted from atrial flutter to sinus rhythm and subsequently reverted back to atrial flutter (Figure 1A). This phenomenon was reproducible (Figure 1B). One hypothesized mechanism for this “cautery-version” was high-frequency energy produced by cautery, coupled onto the leads and to the tip-tissue interface. However, there was no direct contact with either the device or leads, so this seems unlikely. Monopolar cautery can cause leakage of low-frequency (50–60 Hz) current, which is within the range of frequencies to which the myocardium is sensitive (30–110 Hz); this has previously been reported to cause ventricular fibrillation.^1^, ^2^, ^3^ Although to the best of our knowledge not previously described, this mechanism could theoretically convert arrhythmias such as atrial flutter. The positioning of the device in this case could have resulted in the electrocautery preferentially delivering energy to the atria, resulting in the observed phenomenon. There were no apparent adverse clinical consequences in this case. However, awareness of this phenomenon may prompt extra care when using cautery, particularly in patients who have anticoagulation interrupted during pacemaker generator replacement, as this may increase the risk of stroke. If concern for risk of stroke is high, rapid atrial pacing could be used to reinduce atrial fibrillation or flutter if this does not occur spontaneously. Further study is required to further define patient, procedural, and device characteristics that may predispose to this phenomenon.

**Key Teaching Points**

• Electrocautery has been previously reported to rarely induce ventricular arrhythmias.
• In this case, electrocautery resulted in recurrent cardioversion from atrial flutter to sinus rhythm in a patient with a dual-chamber pacemaker undergoing pulse-generator replacement.
• Awareness of this phenomenon may prompt caution when using cautery in patients in whom anticoagulation has been interrupted, as such a cardioversion may increase the risk of cardioembolic stroke.
